# Autistic Traits in Male and Female Students and Individuals with High Functioning Autism Spectrum Disorders Measured by the Polish Version of the Autism-Spectrum Quotient

**DOI:** 10.1371/journal.pone.0075236

**Published:** 2013-09-23

**Authors:** Ewa Pisula, Rafał Kawa, Łukasz Szostakiewicz, Izabela Łucka, Magdalena Kawa, Agnieszka Rynkiewicz

**Affiliations:** 1 Faculty of Psychology, University of Warsaw, Warsaw, Poland; 2 Department of Developmental, Psychotic and Geriatric Psychiatry, Medical University of Gdansk, Gdansk, Poland; Ecole Normale Supérieure, France

## Abstract

So far no standardized screening instrument for autism spectrum disorders for adults has been developed in Poland. The main aim of the study was to explore the properties of the Polish version of the Autism-Spectrum Quotient (AQ), especially its reliability and discriminating power. The second purpose was to establish whether the pattern of sex and area of study differences in the amount of autistic traits found in other countries also exist in Poland. The groups in the study included students (n = 2819), adults with ASD (n = 60) and a non-clinical sample (n = 60) matched with the ASD group for age, sex, education and place of residence. The Polish version of AQ proved to be reliable, although - as in studies conducted in other countries - the internal consistency coefficients for subscales (with exception for social skill) were low. ASD diagnosis was the most powerful determinant of AQ scores. Sex differences in autistic traits and a relationship between autistic traits and area of study were found.

## Introduction

Among the main characteristics of autism spectrum disorders (ASD) are socio-communication difficulties, including problems in establishing social relations: maintaining reciprocal interaction and communication with others, accompanied by restricted patterns of behaviour, interests and activities [1,2]. The extent of difficulties typical of autism varies considerably within the spectrum [3]. Moreover, it has been shown that difficulties similar to those found in individuals with ASD may be present in their close relatives [4,5,6,7,8,9,10]. Those findings support applying the dimensional approach to the study of autistic traits.

The hypothesis that autistic traits may display a continuous distribution throughout the population has been tested in a number of studies [11,12,13]. Research in this area may bring us closer to an understanding of the essence of autism and the mechanisms underlying the emergence of its characteristic symptoms [14,15]. An important step in this process was the development of the Autism-Spectrum Quotient (AQ), an instrument for measuring autistic traits in the general population [16]. This is a brief, self-administered questionnaire, which, as intended by its authors, can be used in screening for ASD in people aged 16 years and older with normal intelligence. It consists of 50 statements referring to five functional domains: social skill, attention switching, attention to detail, communication and imagination. Subjects respond on a 4-point Likert scale to indicate to what extent they agree or disagree with each statement. The overall score is between 0 and 50 points, and the results for the subscales (one for each functional domain) – between 0 and 10 points.

### The diagnostic value of the AQ

Baron-Cohen et al. [16] tested the diagnostic value of the questionnaire by comparing the results of four groups: individuals with Asperger syndrome or High Functioning Autism (AS/HFA, n = 58), controls selected randomly from 500 adults who had completed the AQ (n = 174), students of Cambridge University (n = 840) and winners of the UK Mathematics Olympiad (n = 16). Mean total AQ score in the AS/HFA group was 35.8 versus 17.6 in the student group and 16.4 in the adult controls. The discriminative qualities of the AQ were confirmed in other studies [17,18,19,20,21].

Studies using the AQ have been conducted in a variety of geographical locations and cultural contexts, including Scotland [22,23], Japan [24,25], the Netherlands [13,26,18], the USA [17,27], Canada [28,29], Italy [8] and China [30]. For example, the Japanese study [31] included 3 groups of participants: adults with AS/HFA (n = 57), an adult control group (n = 194) and students (n =1050). The results mostly confirmed those reported by Baron-Cohen [16]. Total AQ in the Japanese AS/HFA sample was 37.9 (compared to 35.8 in the British sample), 18.5 in the control group (compared to 16.4) and 20.7 in the student group (versus 17.6).

In a study conducted by Lombardo et al. [19], mean total AQ score in the group of adults with AS/HFA was 33.93, compared to 16.50 in the control group matched for age, sex and IQ. Woodbury-Smith et al. [21] have shown that the 26+ threshold score displays the best screening properties, yielding 83% of correct diagnoses. Ketelaars et al. [18] reported even lower total AQ and subscale scores for participants with AS/HFA. Mean total AQ in this relatively small sample (n = 15) was 22.5, and for non-ASD participants (n = 21) 21.8. Similarly, in one of the most recent studies, conducted by Bishop and Seltzer [17] on a group of 65 participants with AS, the mean total AQ was 24.62, i.e. lower than in earlier studies. In that particular study only 11 participants (17% of the sample) met the diagnostic cut-off of 32 proposed by Baron-Cohen et al. [16], while 24 (37%) met the screening cut-off of 26 proposed by Woodbury-Smith et al. [21].

### Sex and areas of study differences in autistic traits

In the first study on the use of the AQ questionnaire, Baron–Cohen et al. [16] reported sex differences: mean total AQ score was higher in male students than in female students. Males also scored higher on all AQ subscales, with the exception of attention to detail. The authors interpreted these findings as evidence for the hypothesis that significantly more males than females in the general population demonstrate autistic traits of moderate severity. In the AS/HFA group there were no sex differences either in the total AQ nor in the subscales. This study showed also other interesting differences. Students of science scored higher than humanities and social sciences students, and students of math scored higher than other students of science. These results confirmed the hypothesis that autistic traits may be associated with scientific skills [32].

Similar sex and area of study-based differences in the AQ were found in research conducted in the Netherlands. In the first such study, Hoekstra et al. [13] tested twins aged 18 years, their siblings and their parents. The study employed an alternative scoring method for the questionnaire: instead of scoring responses as 0 or 1, a 1 to 4 points scale was used (1 for “definitely disagree”, 2 for “slightly disagree”, 3 for “slightly agree” and 4 for “definitely agree”). Males scored higher in the total AQ than females. In the parents’ group, the mean score of fathers was also higher than that of mothers. In a subsequent study [26] there were four groups of participants: university students (comprising students of humanities, social sciences and natural/technical sciences (n = 961)), a general population sample (n = 302), 18-year-old twins including their siblings (n = 117) and psychiatric patients (n = 36). A factor analysis of AQ scores yielded two factors: social interaction and attention to detail. For both factors there was a relationship between area of study and AQ scores, as well as sex x area of study interaction. Science students scored higher than students of humanities and social sciences. No effect of sex was found in the analysed faculties, although it was present in the total AQ score as well as in one of the two factors revealed in the factor analysis, namely social interaction. No such effect was found for attention to detail.

Wheelwright et al. [33] and Woodbury-Smith et al. [21] also found no sex differences in AQ scores in the AS/HFA group. In the study cited above conducted by Wakabayashi et al. [31], men scored higher than women in the students, controls and AS/HFA groups, although in the AS/HFA group the difference was not statistically significant. Austin [22] stated that in the general population sample, males scored higher than females in total AQ, social skill, communication and imagination. Thus, sex differences in AQ scores appear to be cross-cultural independent. The effect of sex was confirmed on a large sample of Japanese students by Kunihira et al. [24]. Males scored higher than females in all of the AQ subscales excepting attention to detail: this subscale displayed no main effect of sex.

Not all researchers, however, have confirmed the presence of sex differences in AQ scores. They were not found by Hurst et al. [27,34], and in the [27] study females scored even slightly higher than males in both total AQ and the majority of subscales (except imagination), although the differences were not statistically significant.

The question of sex differences in autistic traits is a common theme in research studies using the AQ questionnaire. According to the Extreme Male Brain Theory of Autism by S. Baron-Cohen [35], the mental functioning of people with autism features a number of typically male brain characteristics at extreme intensity, including poorer empathizing and better systemizing. There is a debate about the apparent disparity in the incidence of autism in males and females [36,37,38]. Exploration of sex differences in the severity of autistic traits in various groups may contribute to a better understanding of the relationship between sex and autism. Moreover, studies in different geographical locations and cultural contexts may provide valuable insight into the nature of sex differences in the context of findings indicating that particular nations may differ in the masculinity-femininity dimension [39,40].

### The present study

To date, no standardized instrument which could be useful in ASD screening purposes has been made available in Poland. The diagnosis of ASD is clinically based, made in accordance with the ICD-10 criteria [2] by a psychiatrist, in cooperation with a clinical psychologist and - depending on the particular characteristics and problems experienced by the individual undergoing diagnosis – occupational therapist, neurologist, pediatrician and educator [41]. The process of adaptation and validation of the standardized methods commonly used around the world in the diagnosis of ASD, such as ADOS-2 [42], ADI-R [43] or SCQ [44] is just beginning. The preparation and validation of the AQ for the Polish-speaking population is important for both scientific and ASD screening purposes. Thus, the first aim of the study was to examine the properties of the Polish version of the AQ, particularly to estimate the internal consistency reliability, test-retest reliability and discriminative value of this questionnaire.

The second aim of the study was to explore the distribution of autistic traits in three samples derived from the Polish population, and to examine whether patterns of sex and areas of study differences reported for autistic traits measured by the AQ in the original British study [16] would be reproduced in Poland. A large sample of Polish students (n = 2819) as well as a smaller high functioning ASD group (n = 60) and controls matched in terms of sex, age, level of education and place of residence (n = 60) were tested using the Polish version of the AQ. The study procedure was modelled primarily on that of Baron-Cohen et al. [16] as well as subsequent studies conducted in other countries [26]. Taking into account that some earlier studies featured somewhat smaller groups of females with AS/HFA (e.g. in the Baron-Cohen et al. [16] study there were 45 males and 13 females, and in the Ketelaars et al. [18] study there were 12 males and only 3 females), and that some researchers made no gender-based distinctions [26], the present study (involving 39 males and 21 women) may provide additional information on sex differences in autistic traits in people with high functioning ASD.

## Methods

### Participants and procedure

The study included 2939 participants belonging to three groups. The first group (Students) was composed of 2819 students from nine institutions of higher education: universities, technical universities, medical universities, colleges of education and higher schools of economics in northern, central and western Poland, aged 18-30 years (M = 20.72, SD = 1.68). The Students group included 1328 males (age: M = 20.33, SD = 1.48) and 1491 females (age: M = 21.07, SD = 1.78). Participants were divided into four subgroups based on their areas of study:

• students of humanities (558 in total, 501 females and 57 males). The group included students of Polish studies, applied linguistics, law, history, French studies, classical studies, Russian studies and modern languages. Mean age of participants was 21.38 years, SD = 1.60 (M = 21.39, SD = 1.62 for females; M = 21.32, SD = 1.48 for males).

• medicine students (428 in total), including 259 females and 169 males. They were attending the faculties of medicine, dentistry and nursing. Mean age of participants was 21.53 years, SD = 2.07 (M = 21.51, SD = 2.16 for females; M = 21.56, SD = 1.94 for males).

• students of natural sciences and engineering (1496 in total), including 492 females and 1004 males. Their faculties included physics, mathematics, biology, biotechnology, food technology, ship construction, engineering (environmental, production), chemistry, mechanical engineering, electronics and telecommunications. Mean age in this group was 20.06 years, SD = 2.22 (M = 20.20, SD = 0.98 for females; M = 19.99, SD = 1.09 for males).

• students of social sciences. This group comprised 337 participants, including 239 females and 98 males. They were students of psychology, pedagogy (general and special education), resocialization and management. Mean age of participants was 21.54 years, SD = 2.22 (M = 21.70, SD = 2.21 for females; M = 21.14, SD = 2.19 for males).

The second group (high functioning ASD group) consisted of 60 high functioning individuals with ASD aged 17-44 years (M = 22.10, SD = 5.87). The group included 39 males (aged 18 to 35 years, M = 20.94, SD = 4.73) and 21 females (aged 18-44 years, M = 24.24, SD = 7.2). Participants were contacted through institutions and organisations providing assistance to individuals with ASD in Poland, as well as psychiatric clinics providing diagnoses of ASD. For each participant from this group the first inclusion criterion was a formal diagnosis of Asperger syndrome (n = 53) or childhood autism (n = 7), issued in accordance with legal regulations applied in Poland by psychiatrists, based on the ICD-10 diagnostic criteria [2]. This diagnosis is made on the basis of a clinical observation and structured interview with parent/caregiver. The interview concerns many topics related to early childhood development, later development and behavioural problems, including age at onset of developmental problems, social skills, language and communication, stereotyped and repetitive patterns of behaviour, sensory problems, the course of motor development and particular interests. Usually, a clinical psychologist is also involved in the diagnostic process. At the time of data collection no validated Polish version of ADOS, ADI-R or SCQ was available, so we could not use them as sources of information on symptoms of ASD in participants. To increase the accuracy of qualification to the ASD group, all of the subjects were assessed for presence of ASD according to ICD-10 criteria by two independent clinicians with experience in diagnosing ASD. Only these individuals who met the diagnostic criteria for ASD in the course of two independent evaluations run by two clinicians were included in the study. Moreover, subjects with IQ below average (IQ < 90) as measured by the Wechsler Adult Intelligence Scale-Revised [45], in Polish adaptation by Brzeziński et al. [46], were excluded. All subjects had successfully completed primary education at mainstream schools (9 years of education). During the study, 24 participants were attending secondary or post-secondary schools (general high schools, technical and vocational secondary schools), 16 were attending universities (areas of study included computer science, physics, finance and medicine), and 11 had graduated. Individuals suffering from depression and substance abuse were excluded. All subjects were Polish native speakers. Seven participants were permanent residents of villages, while the others lived in cities.

The third group (Controls) comprised 60 individuals from the general population (39 males and 21 females) who had not been diagnosed with ASD, selected from a larger group of 396 adults who had completed the AQ. They were matched to the participants in the ASD group in terms of four criteria: sex, age (≤ 6 month difference), level of education and place of residence (village/city). Mean age of participants was 22.57 years (SD = 6.27); for males M = 21.87 (SD = 5.70) and for females M = 24.85 (SD = 7.18).

Ethical approval was granted by an Ethics Committee of the Faculty of Psychology, University of Warsaw, prior to recruiting participants. In case of participants under 18 years of age, written consent from their parents or legal guardians was obtained. Participants aged 18 and above were informed about the voluntary and anonymous character of their participation in the study and about their right not to participate. Obtaining written consent from adult participants in non-invasive studies that involve no manipulation and are anonymous is not required in Poland.

Questionnaires with ≥10% of information missing (i.e. no responses to five or more statements) were treated as incomplete and excluded from analysis. This was the case for 68 questionnaires in the Students group and for two questionnaires in the ASD group. Since the distribution of the missing data across the items in the remaining cases (<10% of information missing) approach random distribution, and the missingness did not exceed 0.5% of the data (521 out of 146 650), the missing values have not been included in the analyses, with no further corrections. The total number of incomplete questionnaires included in the analysis was 226 in Students group (40 missing four items, 50 missing three items, 65 two and 81 one item) and 1 in ASD group (one missing item).

### Instrument

#### The Polish version of the Autism-Spectrum Quotient

The AQ was translated from English into Polish upon consent of Prof. Simon Baron-Cohen by Ewa Pisula, Ph.D., Agnieszka Rynkiewicz, MAT, M.D., and Izabela Łucka, M.D., Ph.D. Next, a different translator translated the Polish version AQ back into English. The original English and back-translated versions were compared by a native English speaker. The text was edited according to the native English speaker comments, following which the reformulated items were translated into English by a translator not familiar with the content of the comments and original questionnaire then compared with the original by a native English speaker; the final Polish version of the AQ was then established (available at www.psychologia.pl/rehabilitacja, as well as from the first author upon request).

The Polish version of the AQ employs the same scoring system as the original. Participants responded to each of 50 statements on a four-point Likert scale (1 “strongly agree”, 2 “slightly agree”, 3 “slightly disagree”, 4 “strongly disagree”). Total AQ, as well as the five subscale scores (social skill, attention switching, attention to detail, communication and imagination) were calculated using a key provided by Simon Baron-Cohen, with one point awarded for an answer consistent with the key (i.e. suggesting an autistic trait) and no point for an inconsistent one (no autistic trait). In 24 statements one point was scored for “strongly agree” or “slightly agree”, and in 26 statements for “strongly disagree” or “slightly disagree” (statements 1, 3, 8, 10, 11, 14, 15, 17, 24, 25, 27, 28, 29, 30, 31, 32, 34, 36, 37, 38, 40, 44, 47, 48, 49, 50). The total result was the sum of all points scored by the participant, ranging from 0 to 50. Results in each of the five subscales ranged from 0 to 10 points.

## Results

Data analysis involved several stages, including analysis of the reliability of AQ, distributions of the AQ scores, discriminating power group comparisons and items analysis. The statistical tests used in subsequent stages of the analyses are described in the appropriate parts of the Results section. The analyses were performed using the Tanagra software [47]. Table 1 provides descriptive statistics of results obtained in the AQ by males and females from Students, the ASD group and Controls.

**Table 1:** Means and standard deviations of total AQ and five subscales of the AQ scores for males and females in the Students, ASD group and Controls.

Group and Sex	N	Total AQ M(SD)	Social skill M(SD)	Attention switching M(SD)	Attention to detail M(SD)	Communi-cation M(SD)	Imagination M(SD)
Students							
Total	2819	16.64 (5.62)	1.94 (2.06)	4.66 (2.19)	5.42 (2.15)	2.02 (1.71)	2.60 (1.96)
Males	1328	18.13 (5.79)	2.25 (2.25)	4.84 (2.00)	5.48 (2.12)	2.42 (1.84)	3.14 (1.80)
Females	1491	15.31 (5.11)	1.66 (1.83)	4.51 (1.96)	5.36 (2.17)	1.66 (1.49)	2.12 (1.57)
ASD							
Total	60	30.73 (8.64)	6.00 (2.61)	7.22 (2.42)	6.23 (2.27)	5.68 (2.78)	5.52 (2.43)
Males	39	29.28 (8.69)	5.69 (2.68)	6.92 (2.42)	6.26 (2.21)	5.05 (2.76)	5.36 (2.65)
Females	21	33.43 (8.04)	6.57 (2.44)	7.76 (2.36)	6.19 (2.44)	6.86 (2.48)	5.81 (1.99)
Controls							
Total	60	16.72 (6.45)	2.45 (1.99)	4.12 (2.15)	3.95 (2.02)	3.12 (2.26)	3.08 (2.03)
Males	39	17.15 (6.03)	2.74 (1.98)	4.33 (1.92)	4.10 (1.98)	3.15 (2.21)	3.10 (2.14)
Females	21	15.90 (7.24)	2.43 (2.13)	3.71 (2.26)	3.67 (2.11)	3.05 (2.42)	3.05 (1.86)

M – Mean; SD – Standard Deviation

### Internal Consistency and Test-Retest Reliability

The internal consistency of the AQ was calculated using Cronbach’s α coefficient in the total sample. The results were thus: for total AQ = .71; social skill = .71; attention switching = .49; attention to detail = .60; communication = .50; imagination = .45. The Cronbach’s α coefficients for the total AQ scores were also assessed for each group in the study and were as follows: in the Students sample: .66, in the ASD sample: .86 and in the control sample: .75. The internal consistiencies for the subscale were estimated at: α = .70/.72/.60 (social skill), α = .62/.76/.64 (communication), α = 57/.60/.57 (attention to detail), α = .53/.73. /53 (attention switching) and α = .55/.65/.54 (imagination).

In order to assess the test-retest reliability the study was conducted on a group of 80 students. After a 1-2 month time interval the measurement was repeated. Sixty eight participants returned the AQ the second time (85%). Scores from the first and second AQ measurements did not differ statistically (t = 0.39, df = 67, p = 0.7). The Pearson r coefficients were as follows: r = .76 for total AQ, r = .78 for social skill, r = .62 for attention to detail, r = .65 for attention switching, r = .72 for communication and r = .67 for imagination.

### Discriminating power of items

In order to estimate the discriminating power of items in the Polish version of AQ, the point-biserial coefficient (r_pb_) was used [48]. The results of this analysis are presented in Table 2.

**Table 2:** The discriminating power of items (n = 2939).

**Scale**	**Item #**	**r_pb_**
Social skill	1	.530
	11	.656
	13	.505
	15	.586
	22	.601
	36	.424
	44	.666
	45	.479
	47	.658
	48	.454
Attention switching	2	.428
	4	.288
	10	.465
	16	.283
	25	.446
	32	.511
	34	.497
	37	.473
	43	.429
	46	.532
Attention to detail	5	.423
	6	.559
	9	.404
	12	.514
	19	.470
	23	.461
	28	.464
	29	.472
	30	.358
	49	.467
Communication	7	.470
	17	.527
	18	.322
	26	.574
	27	.434
	31	.335
	33	.498
	35	.356
	38	.590
	39	.492
Imagination	3	.359
	8	.452
	14	.465
	20	.425
	21	.395
	24	.352
	40	.538
	41	.339
	42	.439
	50	.553

As shown in Tab. 2, the r_pb_ value of almost all items was > .3, and in two items (4 and 16) it was >.28. Thus, it can be assumed that discriminating power of each item is acceptable.

Moreover, the percentages of subjects with ASD and Controls scoring a point in each AQ item were calculated (Table 3). Only for item 29 (*I am not very good at remembering phone numbers*) was the percentage of subjects with ASD and Controls scoring on it the same. As for all the other items, the percentage of people with ASD was higher than Controls. This indicates that AQ was useful in distinguishing participants with ASD from Controls in this study.

**Table 3:** Percentages of ASD group and Controls who scored in individual items of the AQ.

AQ Item	ASD (n = 60)	Controls (n = 60)
1	65.00	25.00
2	81.67	46.67
3	23.33	20.00
4	76.67	43.33
5	63.33	38.33
6	65.00	31.67
7	56.67	38.33
8	33.33	25.00
9	53.33	23.33
10	63.33	28.33
11	51.67	13.33
12	90.00	41.67
13	38.33	30.00
14	50.00	35.00
15	63.33	21.67
16	85.00	60.00
17	55.00	21.67
18	58.33	41.67
19	53.33	23.33
20	58.33	13.33
21	46.67	31.67
22	80.00	35.00
23	66.67	61.67
24	58.33	25.00
25	73.33	40.00
26	73.33	43.33
27	63.33	30.00
28	63.33	43.33
29	45.00	45.00
30	58.33	48.33
31	46.67	20.00
32	71.67	43.33
33	51.67	26.67
34	58.33	25.00
35	43.33	31.67
36	53.33	31.67
37	56.67	23.33
38	61.67	31.67
39	58.33	26.67
40	70.00	40.00
41	75.00	41.67
42	56.67	36.67
43	75.00	51.67
44	50.00	15.00
45	71.67	30.00
46	80.00	50.00
47	53.33	10.00
48	70.00	33.33
49	65.00	38.33
50	76.67	40.00

### Distribution of AQ scores and determining a useful cut-off

The range of total AQ scores in the ASD group was from 11 to 48 points, in the Control group from 6 to 35 points, and in the Students group from 2 to 40 points.

**Figure 1:**
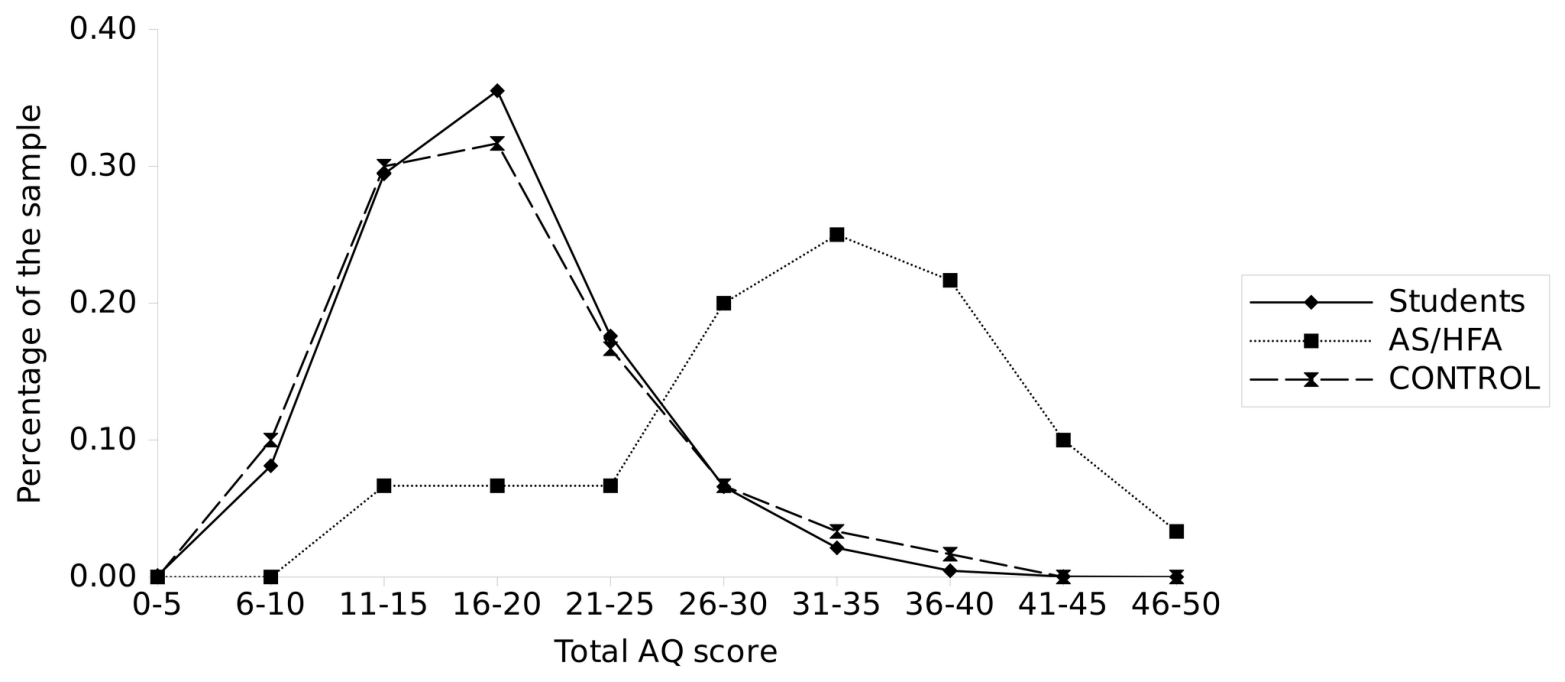
Distribution of total AQ scores in the ASD group, Controls and Students.

The distribution of total AQ scores by group is presented in Figure 1. In order to establish a useful cut-off that would discriminate the groups with as many true positives and as few false positives as possible, the distribution of cumulative rates of individual total AQ values for the ASD group, Controls and Students was analyzed (Tab. 4). As shown in Tab. 4, 32+ points (cut-off in the British study) were scored by 53.33% of participants in the ASD group, 5% in the Control group and 1.56% in the Students group. As much as 80% of the ASD group scored ≥ 25 points, which compares to less than 11.7% of the Control group and 9.22% of the Students group. This cut-off seems to have better screening properties for females (respectively: 86%, 10%, 5%) than males (72%, 13%, 14%). For females, the best score for distinguishing females with ASD from Controls and Students is a score of 28+ (respectively: 76%, 3%, 1.88%), while for males its screening properties are weaker (54%, 10%, 7.6%).

**Table 4:** Cumulative rates of AS/HFA, Controls and Students scoring at or above each AQ score.

AQ score	ASD group (n = 60)	ASD males (n = 39)	ASD females (n = 39)	Controls (n = 60)	Control males (n = 39)	Control females (n = 21)	Students group (n = 2819)	Students males (n = 1491)	Students females (n = 1328)
0	100.00	100.00	100.00	100.00	100.00	100.00	100	100	100
1	100.00	100.00	100.00	100.00	100.00	100.00	100	100	100
2	100.00	100.00	100.00	100.00	100.00	100.00	100	100	100
3	100.00	100.00	100.00	100.00	100.00	100.00	99.96	100	99.93
4	100.00	100.00	100.00	100.00	100.00	100.00	99.93	100	99.87
5	100.00	100.00	100.00	100.00	100.00	100.00	99.89	99.92	99.87
6	100.00	100.00	100.00	100.00	100.00	95.00	99.50	99.55	99.46
7	100.00	100.00	100.00	98.33	95.00	95.00	98.94	99.47	98.46
8	100.00	100.00	100.00	95.00	95.00	90.00	96.81	98.72	95.10
9	100.00	100.00	100.00	93.33	92.00	86.00	94.89	97.59	92.49
10	100.00	100.00	100.00	90.00	85.00	81.00	91.77	95.63	88.33
11	100.00	97.00	100.00	83.33	82.00	71.00	87.48	93.00	82.56
12	98.33	95.00	100.00	78.33	74.00	67.00	82.05	88.86	75.99
13	96.67	92.00	100.00	71.67	69.00	62.00	76.45	84.79	69.01
14	95.00	90.00	100.00	66.67	67.00	48.00	69.71	79.29	61.17
15	93.33	90.00	100.00	60.00	59.00	38.00	62.33	72.89	52.92
16	93.33	90.00	95.00	51.67	54.00	38.00	53.88	64.38	44.53
17	91.67	90.00	95.00	48.33	44.00	24.00	45.90	55.27	37.56
18	91.67	85.00	90.00	36.67	38.00	24.00	39.80	49.70	30.99
19	86.67	85.00	90.00	33.33	31.00	24.00	33.63	44.20	24.21
20	86.67	85.00	90.00	28.33	26.00	19.00	26.82	35.32	19.25
21	86.67	85.00	86.00	23.33	23.00	14.00	22.53	30.65	15.29
22	85.00	82.00	86.00	20.00	15.00	14.00	17.06	23.57	11.27
23	83.33	79.00	86.00	15.00	13.00	10.00	13.98	20.18	8.45
24	81.67	77.00	86.00	11.67	13.00	10.00	11.60	17.47	6.37
25	80.00	72.00	86.00	11.67	13.00	10.00	9.22	13.93	5.03
26	76.67	69.00	81.00	11.67	10.00	10.00	7.34	10.77	4.29
27	73.33	62.00	76.00	10.00	10.00	8.00	5.78	9.26	2.68
28	66.67	54.00	76.00	8.33	10.00	3.00	4.58	7.61	1.88
29	61.67	54.00	71.00	5.00	10.00	3.00	3.65	6.25	1.34
30	60.00	51.00	71.00	5.00	10.00	3.00	2.63	4.59	0.87
31	58.33	46.00	67.00	5.00	10.00	3.00	1.81	3.01	0.74
32	53.33	38.00	62.00	5.00	5.00	0.00	1.56	2.71	0.54
33	46.67	26.00	62.00	1.67	5.00	0.00	1.06	1.96	0.27
34	38.33	21.00	62.00	1.67	5.00	0.00	0.74	1.28	0.27
35	35.00	18.00	57.00	1.67	0.00	0.00	0.50	0.83	0.20
36	31.67	18.00	43.00	0.00	0.00	0.00	0.35	0.60	0.13
37	26.67	15.00	29.00	0.00	0.00	0.00	0.25	0.53	0
38	20.00	13.00	24.00	0.00	0.00	0.00	0.04	0.08	0
39	16.67	10.00	19.00	0.00	0.00	0.00	0.04	0.08	0
40	13.33	10.00	19.00	0.00	0.00	0.00	0.04	0.08	0
41	13.33	8.00	10.00	0.00	0.00	0.00	0	0	0
42	8.33	8.00	10.00	0.00	0.00	0.00	0	0	0
43	8.33	5.00	10.00	0.00	0.00	0.00	0	0	0
44	6.67	3.00	5.00	0.00	0.00	0.00	0	0	0
45	3.33	3.00	0.00	0.00	0.00	0.00	0	0	0
46	1.67	3.00	0.00	0.00	0.00	0.00	0	0	0
47	1.67	3.00	0.00	0.00	0.00	0.00	0	0	0
48	1.67	0.00	0.00	0.00	0.00	0.00	0	0	0
49	0.00	0.00	0.00	0.00	0.00	0.00	0	0	0
50	0.00	0.00	0.00	0.00	0.00	0.00	0	0	0

### Group and sex differences on the AQ

A two-way ANOVA was used to compare the scores of males and females in the study group. The first factor, Group, assumed one of three values: Students, ASD group and Controls, and the second factor was Sex. To avoid Type I error, a Bonferroni correction was applied. Therefore, the alpha level was set at 0.001 for all analyses. Results are shown in Tab. 5. Post-hoc Scheffe statistics showed that the ASD group scored higher than the other groups for all measured variables. Group effect size was significantly larger than the size of the remaining effects for total AQ (η^2^= .11) and for the subscales. The only exception was imagination, where the effect of sex was slightly larger than the effect of group. As far as attention to detail, there was only an effect of group. There were no differences in total AQ, social skill, attention switching and imagination between Controls and Students. There were differences in attention to detail (Students scored higher) and communication (Controls scored higher). The interaction group x sex effects were so weak that we abandoned efforts at their interpretation.

**Table 5:** Two-way ANOVA group x sex over the total AQ and AQ subscales.

Variables	F	p	η^2^_(partial)_	Source of effect
Total AQ	F(2,2934) = 177.481	< .001	.11	main effect of Group
	F(1,2934) = 193.429	< .001	0.06	main effect of Sex
	F(2,2934) = 11.041	< .001	0.007	interaction effect of Group x Sex
Social skill	F(2,2934) = 109.991	< .001	.07	main effect of Group
	F(1,2934) = 66.238	< .001	0.02	main effect of Sex
Attention switching	F(2,2934) = 49.30	< .001	.03	main effect of Group
	F(1,2934) = 21.413	< .001	0.007	main effect of Sex
Attention to detail	F(2,2934) = 18.477	< .001	.012	main effect of Group
Communication	F(2,2934) = 134.407	< .001	.08	main effect of Group
	F(1,2934) = 147.867	< .001	0.05	main effect of Sex
	F(2,2934) = 16.068	< .001	0.01	interaction effect of Group x Sex
Imagination	F(2,2934) = 75.799	< .001	.05	main effect of Group
	F(1,2934) = 257.444	< .001	0.08	main effect of Sex
	F(2,2934) = 6.923	< .001	0.005	interaction effect of Group x Sex

The main effect of sex was revealed for five analysed variables: total AQ (males: M = 18.42, SD = 6.17; females: M = 15.57, SD = 5.60), social skill (males: M = 2.35, SD = 2.33; females: M = 1.74, SD = 1.93), attention switching (males M = 4.88, SD = 2.04; females M = 4.54, SD = 2.01), communication (males: M = 2.52, SD = 1.93; females: M = 1.75, SD = 1.65) and imagination (males: M = 3.20, SD = 1.87; females: M = 2.19, SD = 1.64).

In the Students group, males scored higher than females in five analysed variables: total AQ (t(2819) = -13.63, p < .001), social skill (t(2819) = -7.61, p < .001), attention switching (t(2819) = -4.41, p < .001), communication (t(2819) = -11.99, p < .001) and imagination (t(2819) = -15.86, p < .001). The difference for attention to detail was not significant.

The only statistically significant difference between males and females in the ASD group was found for communication (t(60) = 2.59, p < .05), in which males scored higher than females. In the Controls there were no differences between the results of males and females, although mean scores in total AQ and subscales were slightly higher for males than females.

### Areas of study differences in Students group

Descriptive statistics for Students of different faculties are presented in Tab. 6. The results for males and females of different areas of study were compared to the total AQ and five AQ subscales using a one-way ANOVA. The factor, area of study, had four values: humanities, medicine, science and social science. As in the initial analyses, to avoid Type I error, a Bonferroni correction was applied and the alpha level was set at 0.001. The results of the analyses are shown in Tab. 7. As seen in the data in Tab. 7, area of study effects were present for total AQ and all subscales. Post-hoc Scheffe tests revealed that science students scored higher than all other groups in total AQ and imagination, and higher than social science and medicine students in social skill, attention switching, attention to detail and communication. The humanities students scored higher than medicine and social science students in all measured variables except imagination. It should be noted, however, that the area of study factor accounted for only 2% to 7% of variance in the measured variables.

**Table 6:** Means and standard deviations by area of study.

Area of study and sex	N	Total AQ score M(SD)	Social skill M(SD)	Attention switching M(SD)	Attention to detail M(SD)	Communi-cation M(SD)	Imagination M(SD)
Humanities	558	16.20 (5.17)	2.08 (2.10)	5.00 (2.03)	5.35 (2.14)	1.85 (1.54)	1.92 (1.54)
Medicine	428	14.34 (5.38)	1.55 (1.69)	4.33 (1.88)	4.45 (2.27)	1.55 (1.63)	2.46 (1.60)
Science	1496	17.93 (5.73)	2.16 (2.23)	4.74 (1.99)	5.73 (2.07)	2.33 (1.80)	2.97 (1.80)
Social science	337	14.63 (4.51)	1.23 (1.37)	4.19 (1.91)	5.39 (1.95)	1.51 (1.35)	2.30 (1.68)

M – Mean; SD – Standard Deviation

**Table 7:** One way ANOVA (area of study as a factor) over the total AQ and AQ subscales.

Variables	F	p	η^2^_(partial)_	Source of effect
Total AQ	F(3,2818) = 70.037	< .0001	.07	Sci > Soc, Sci > Med, Sci > Hum, Hum > Med, Hum > Soc
Social skill	F(3,2818) = 25.153	< .0001	.03	Sci > Soc, Sci > Med, Hum > Soc, Hum > Med
Attention switching	F(3,2818) = 16.769	< .0001	.02	Sci > Soc, Sci > Med, Hum > Soc, Hum > Med
Attention to detail	F(3,2818) = 41.284	< .0001	.04	Sci > Med, Sci > Hum, Soc > Med, Hum > Med
Communication	F(3,2818) = 40.613	< .0001	.04	Sci > Soc, Sci > Med, Sci > Hum, Hum > Soc
Imagination	F(3,2818) = 57.28	< .0001	.06	Sci > Soc, Sci > Med, Sci > Hum, Med > Hum, Soc > Hum

Hum – humanities, Med – Medicine, Sci – Science, Soc – Social science

## Discussion

Our first goal was to determine the basic psychometric properties of the Polish version of AQ, especially its internal consistency, test-retest reliability and discriminating power of items. We also studied the distribution of AQ scores in the ASD group, Controls and Student groups. Moreover, analysis of sex and areas of study differences in AQ were conducted.

With reference to internal reliability, the results of this investigation are similar to those obtained by other researchers in other language samples. Internal consistency was satisfactory for total AQ (.71) and social skill (.71), while other scales demonstrated lower Cronbach’s α coefficients (from .60 for attention to detail to .45 for imagination). The internal consistency coefficients for subscales in the original study by Baron-Cohen et al. [16] were as follows: communication = 0.65; social skill = 0.77; imagination = 0.65; attention to detail = 0.63; attention switching = 0.67. Our findings showing variability in the level of internal consistency for subscales are very consistent with the results obtained by Hurst et al. [27], who demonstrated that Cronbach’s α coefficients were the highest for total AQ and social skill (.67 and .66 respectively), but lower in the case of attention to detail (.60), communication (.47), attention switching (.41) and imagination (.40). Ingersoll et al. [49] also reported the highest Cronbach’s α coefficient values for total AQ and social skill (.72 and .67 respectively). The values were lower for the other subscales, with the lowest Cronbach’s α found for imagination (.45, the same as in our study for the whole sample). Thus, it may be stated that total AQ and the social skill subscale are characterized by satisfactory reliability, but the reliability of other subscales seems to be clearly lower, regardless of the version of the questionnaire. It is also characteristic that the imagination subscale has the lowest internal reliability in all cited studies. It should be stressed, however, that in the present study the Cronbach’s α coefficients were the highest in the ASD sample (for total AQ: .86, for social skill: .72, for communication: .76, for attention to detail: .60, for attention switching: .73 and for imagination: .65). This may show that AQ is relatively well suited to conducting studies on this group of people, although the internal reliability of four subscales should be improved.

The test-retest reliability of the Polish version of AQ was high for total AQ, social skill and communication (> .70), while slightly lower for the other subscales (but not lower than .62). It should be noted that this measurement, as in the study by Baron-Cohen et al. [16], was conducted only in the Student group. This portion of the analyses provides support for accepting the reliability of the Polish version of AQ, as sufficient.

The next step in our analysis was to test the discriminating power of AQ items. Point-biserial coefficients were calculated, and the results of this analysis showed that the discriminating power of each item was acceptable. Moreover, the percentage of the ASD group and Controls scoring on each item was estimated. In all cases but one, the percentage of subjects with ASD scoring was higher than in the Control group. It seems important that in none of the AQ items was the percentage of subjects whose responses suggested the presence of autistic traits lower in the ASD group than in the control group. The complete lack of differences in the distribution of responses to item 29 (*I am not very good at remembering phone numbers*) is likely to be associated with the standard functionality of modern phones, which can store large numbers of contacts and select them without the need for the user to enter individual digits. Consequently, both individuals with ASD as well as their typically developing counterparts have significantly less experience with memorizing phone numbers than was the case in the past.

In our study sample, the cut-off of 32+ points proposed by Baron-Cohen et al. [16] was reached by 53.33% of participants in the ASD group, 5% in the Control group and only 1.56% in the Students group. Thus, the cut-off properties were slightly different in terms of screening power than in the UK sample, where this score was reached by 79.3% of individuals with ASD and 2.3% of Controls. In the Polish study the number of false negatives was much higher. The 26+ cut-off proposed by Woodbury-Smith et al. [21] was reached by 76.67% of ASD participants in our study, 11.67% of Controls and 7.34% of Students. It appears that relatively good screening effectiveness in Poland would be achieved at a 25+ cut-off, which was reached by 80% of participants with ASD, 11.67% of the Control group and 9.6% of the Students group. This threshold is promising both in the case of females (reached by 86%) and males (72%). Unfortunately, using a score of 25+ as a cut-off may provide too high a percentage of false positives. Moreover, in the study by Baron-Cohen et al. [16], a score of 32+ similarly distinguished females and males with ASD from Controls and Students. In the present study a better cut off for females is a score of 28+ (76% females of the ASD sample scored at this level, 3% of females from the Control group and less than 2% of females from the Students group). Effectiveness was lower in the case of males. The score of 28+ was reached by just 54% of males with ASD, which shows a reasonable risk of false negative assessment. Still, in order to establish a valid cut-off score, further testing is needed on a larger sample of high-functioning individuals with ASD, and the results need to be compared with other clinical populations.

Mean total AQ in the Polish ASD group was 30.73, which is lower than the 35.8 reported in the Baron-Cohen et al. study [16]. The range of total AQ in the ASD group in the present study was 11-48 points, compared to 19-48 points in the aforementioned UK study. Participants with ASD self-reporting in the AQ in the Polish sample also scored lower in comparison to data reported by Meng-Chuan et al. [50], in which males achieved mean total AQ of 32.8 and females of 37.6, as well as the Japanese study, where mean total AQ was 37.9 [31]. In contrast, mean total AQ in our ASD group was higher than in the research conducted by Ketelaars et al. [18] (M = 22.5) and Bishop and Seltzer [17] (M = 24.62). Thus, the impact of cultural factors on the size of total AQ cannot be excluded, but it would be difficult to identify them with any precision on the basis of currently available data.

Although the Students group provides valuable data for sex and area of study differences in AQ scales, due to its age and level of education homogeneity it may not serve as a valid control group for the ASD sample. The present study provides more valid information about the discriminant power of the AQ obtained from comparison of the ASD group with the Controls matched for age, sex, level of education and place of residence. The differences between the Controls and Students in attention to detail (Students scored higher) and communication (Controls scored higher) can probably be attributed to the greater heterogeneity of Controls in terms of factors such as level of education and age. It should be noted that there were no differences between the two groups in terms of total AQ, nor in social skill, attention switching and imagination.

A two-way ANOVA (group x sex) yielded the main effect of group with respect to AQ and five subscales: social skill, attention switching, attention to detail, communication and imagination. The ASD group scored higher than other groups in all these subscales. In addition, the size of the group effect for total AQ was greater than the size of the sex effect, as well as than the group x sex interaction effect. The effect of group was also the largest for each of the subscales, with the exception of imagination.

The results also suggesting a greater severity of autistic traits in high functioning individuals with ASD compared to the general population never diagnosed with ASD and the large sample of students are consistent with expectations. Similar differences have previously been reported [16,18]. In the present study, the most important information about the discriminant power of the AQ was derived from comparison of the ASD group with the Controls matched for age, sex, level of education and place of residence. The differences between the Controls and Students in attention to detail (Students scored higher) and communication (Controls scored higher) can probably be attributed to the disproportion in the sizes of these groups and relatively small size of the Control group (n = 60 versus n = 2819 in the Students group), as well as its greater heterogeneity in terms of factors such as level of education. It should be noted that there were no differences between the two groups in terms of total AQ, nor in social skill, attention switching and imagination.

Analysis of sex differences demonstrated that in total AQ score as well as in four subscales (social skill, attention switching, communication and imagination), male participants scored higher than females, suggesting a greater severity of autistic traits. The only exception was the attention to detail subscale, where no sex differences were found. Exactly the same effects were found by Kunihira et al. [24]. In their study, males scored higher than females in all of the AQ subscales but attention to detail. In the Students group, the largest in our study, the mean total AQ score for males was 18.13, compared to 15.31 for females. Baron-Cohen et al. [16] reported the same direction of differences: in the group of male students the total AQ score was 18.6 versus 16.4 in females.

Analysis of the ASD group brought some interesting results with respect to sex differences. There was one statistically significant difference between the scores of males and females: males scored higher in the communication subscale than females. In both the female and male groups the range of AQ scores was wide, but females had a slightly higher mean score (M = 33.43) than males (M = 29.28); the difference, however, was not statistically significant. Most other researchers investigating autistic traits in females and males with ASD found no differences between these groups [16] [17,31,33,21]. In contrast, Meng-Chuan et al. [50] in their comparison of 33 males and 29 females with diagnosed AS found that females reported more autistic traits (M = 37.6) than males (M = 32.8). The difference was present in self-reported data, even though there were no gender-based differences in childhood ADI-R scores, and ADOS females demonstrated fewer socio-communication symptoms. Thus it seems clear that the question of sex differences in autistic traits severity in people with ASD requires further research, since it is possible that women perceive more differences between their own functioning and that of their typically developing peers, or that they have better insight into their difficulties. Another possibility, as mentioned by Meng-Chuan et al. [50], is that developmental trajectories of women with ASD differ from those of their male counterparts.

The relationship between the severity of autistic trait and area of study was also confirmed by our findings. Science students scored higher than students of medicine and social sciences in total AQ, and higher than students of humanities in attention to detail and imagination. Results indicating a higher severity of autistic traits in science students are consistent with findings reported by other researchers [16,24]. Unexpectedly, in most subscales students of humanities scored higher than students of social sciences and/or medicine (except for imagination). Perhaps the result was influenced by the large presence of students of classical studies and applied linguistics among the humanities students. In contrast to social sciences and medical science students, they might demonstrate some traits common for autism conditions, e.g. the tendency for in-depth, detailed analysis of material (in their case text). The propensity for systemizing typical of autism conditions [35] can also be pronounced in some students of applied linguistics, who may be interested in language as a system.

In conclusion, the results of the study confirm that the Polish version of the AQ has comparable psychometric qualities to other language versions of the questionnaire. It seems to be a promising screening tool in populations of adults in a normal intelligence range as far as the reliability for total AQ and social skill subscale are concerned. The test-retest reliability and discriminating power of items are also satisfactory. Further analysis is required to determine the optimal cut-off score. Sex differences and differences between areas of study were also confirmed with respect to AQ. Some interesting data was obtained on sex-based differences in the severity of self-reported autistic traits in individuals with ASD diagnosis. That last phenomenon should certainly be taken up in a study on a larger sample from the ASD population with stricter control of variables (such as IQ and co-morbidity).

## References

[1] American Psychiatric Association (2004) Diagnostic and Statistical Manual for Mental Disorders, 4th Ed., Text R evision(DSM-IVTR). Washington: American Psychiatric Press.

[2] World Health Organization (2002) Manual of the International Statistical Classification of the Diseases, and Related Health Problems ed. 10, vol. 1; Genewa WHO. 184 p.

[3] Baron-Cohen S (1997) Mindblindness: An Essay on Autism and Theory of Mind. Cambridge, MH. The MIT Press. 171pp.

[4] Bailey A, Palferman S, Heavey L, Le Couteur A (1998) Autism: The phenotype in relatives. J Autism Dev Disord 28: 369–392. doi:10.1023/A:1026048320785. PubMed: 9813774. 9813774 10.1023/a:1026048320785

[5] Bishop DVM, Maybery M, Maley A, Wong D, Hill W et al. (2004) Using self-report to identify the broad phenotype in parents of children with autistic spectrum disorders: A study using the Autism-Spectrum Quotient. J Child Psychol Psychiatry 45: 1431–1436. doi:10.1111/j.1469-7610.2004.00325.x. PubMed: 15482503. 15482503 10.1111/j.1469-7610.2004.00849.x

[6] Constantino JN, Lajonchere C, Lutz M, Gray T, Abbacchi A et al. (2006) Autistic social impairment in the siblings of children with pervasive developmental disorders. Am J Psychiatry 163: 294–296. doi:10.1176/appi.ajp.163.2.294. PubMed: 16449484. 16449484 10.1176/appi.ajp.163.2.294

[7] Piven J, Palmer P, Jacobi D, Childress D, Arndt S (1997) Broader autism phenotype: Evidence from a family history study of multiple-incidence autism families. Am J Psychiatry 154: 185–190. PubMed: 9016266. 9016266 10.1176/ajp.154.2.185

[8] Ruta L, Mazzone D, Mazzone L, Wheelwright S, Baron-Cohen S (2012) The Autism Spectrum Quotient – Italian version: A cross-cultural confirmation of the broader autism phenotype. J Autism Dev Disord 42: 625-633. doi:10.1007/s10803-011-1290-1. PubMed: 21626054. 21626054 10.1007/s10803-011-1290-1

[9] Scheeren AM, Stauder JEA (2008) Broader Autism Phenotype in parents of autistic children: Reality or myth? J Autism Dev Disord 38: 276–287. doi:10.1007/s10803-007-0389-x. PubMed: 17588199. 17588199 10.1007/s10803-007-0389-x

[10] Wheelwright S, Auyeung B, Allison C, Baron-Cohen S (2010) Defining the broader, medium and narrow autism phenotype among parents using the autism spectrum quotient (AQ). Molecular Autism 1: 1-9. doi:10.1186/2040-2392-1-1. PubMed: 20678244. 20678260 10.1186/2040-2392-1-10 PMC2913943

[11] Constantino JN, Todd RD (2003) Autistic traits in the general population: A twin study. Arch Gen Psychiatry 60: 524–530. doi:10.1001/archpsyc.60.5.524. PubMed: 12742874. 12742874 10.1001/archpsyc.60.5.524

[12] Constantino JN, Todd RD (2005) Intergenerational transmission of subthreshold autistic traits in the general population. Biol Psychiatry 57: 655–660. doi:10.1016/j.biopsych.2004.12.014. PubMed: 15780853. 15780853 10.1016/j.biopsych.2004.12.014

[13] Hoekstra RA, Bartels M, Verweij CJH, Boomsma DI (2007) Heritability of autistic traits in the general population. Arch Pediatr Adolesc Med 161: 372–377. doi:10.1001/archpedi.161.4.372. PubMed: 17404134. 17404134 10.1001/archpedi.161.4.372

[14] Robinson EB, Koenen KC, McCormick MC, Munir K, Hallett V et al. (2011) Evidence that autistic traits show the same etiology in the general population and at the quantitative extremes (5%, 2.5%, and 1%). Arch Gen Psychiatry 68: 1113-1121. doi:10.1001/archgenpsychiatry.2011.119. PubMed: 22065527. 22065527 10.1001/archgenpsychiatry.2011.119 PMC3708488

[15] Sucksmith E, Roth I, Hoekstra RA (2011) Autistic traits below the clinical threshold: re-examining the broader autism phenotype in the 21st century. Neuropsychol Rev 21: 360-389. doi:10.1007/s11065-011-9183-9. PubMed: 21989834. 21989834 10.1007/s11065-011-9183-9

[16] Baron-Cohen S, Wheelwright S, Skinner R, Martin CE (2001) The Autism Spectrum Quotient (AQ): Evidence from Asperger syndrome/high functioning autism, males and females, scientists and mathematicians. J Autism Dev Disord 31: 5–17. doi:10.1023/A:1005653411471. PubMed: 11439754. 11439754 10.1023/a:1005653411471

[17] Bishop SL, Seltzer MM (2012) Self-reported autism symptoms in adults with Autism Spectrum Disorders. J Autism Dev Disord 42: 2354-2363. doi:10.1007/s10803-012-1483-2. PubMed: 22361924. 22361924 10.1007/s10803-012-1483-2 PMC3475727

[18] Ketelaars C, Horwitz E, Sytema S, Bos J, Wiersma D et al. (2008) Brief report: Adults with mild autism spectrum disorders (ASD): Scores on the autism spectrum quotient (AQ) and comorbid psychopathology. J Autism Dev Disord 38: 176–180. doi:10.1007/s10803-007-0358-4. PubMed: 17385086. 17385086 10.1007/s10803-007-0358-4 PMC2175022

[19] Lombardo MV, Barnes JL, Wheelwright SJ, Baron-Cohen S (2007) Self-referential cognition and empathy in autism. PLOS ONE 2: 1-11. PubMed: 17849012. 10.1371/journal.pone.0000883 PMC1964804 17849012

[20] Sizoo BB, van den Brink W, Gorissen-van Eenige M, Koeter MW, van Wijngaarden-Cremers PJM et al. (2009) Using the Autism-Spectrum Quotient to discriminate Autism Spectrum Disorder from ADHD in adult patients with and without comorbid substance use disorder. J Autism Dev Disord 39: 1291–1297. doi:10.1007/s10803-009-0743-2. PubMed: 19396535. 19396535 10.1007/s10803-009-0743-2 PMC2727364

[21] Woodbury-Smith MR, Robinson J, Wheelwright S, Baron-Cohen S (2005) Screening adults for Asperger syndrome using the AQ: A preliminary study of its diagnostic validity in clinical practice. J Autism Dev Disord 35: 331–335. doi:10.1007/s10803-005-3300-7. PubMed: 16119474. 16119474 10.1007/s10803-005-3300-7

[22] Austin EJ (2005) Personality correlates of the broader autism phenotype as assessed by the autism spectrum quotient (AQ). Pers Individ Dif 38: 451–460. doi:10.1016/j.paid.2004.04.022.

[23] Stewart ME, Austin E (2009) The structure of the Autism-Spectrum Quotient (AQ): Evidence from a student sample in Scotland. Pers Individ Dif 47: 224-228. doi:10.1016/j.paid.2009.03.004.

[24] Kunihira Y, Senju A, Dairoku H, Wakabayashi A, Hasegawa T (2006) ‘Autistic’ traits in non-autistic Japanese populations: Relationships with personality traits and cognitive ability. J Autism Dev Disord 36: 553-566. doi:10.1007/s10803-006-0094-1. PubMed: 16602034. 16602034 10.1007/s10803-006-0094-1

[25] Kurita H, Koyama T, Osada H (2005) Autism spectrum quotient – Japanese version and its short forms for screening normally intelligent persons with pervasive developmental disorders. Psychiatry Clin Neurosci 59: 490–496. doi:10.1111/j.1440-1819.2005.01403.x. PubMed: 16048456. 16048456 10.1111/j.1440-1819.2005.01403.x

[26] Hoekstra RA, Bartels M, Cath DC, Boomsma DI (2008) Factor structure, reliability and criterion validity of the Autism-Spectrum Quotient (AQ): A study in Dutch population and patient groups. J Autism Dev Disord 38: 1555–1566. doi:10.1007/s10803-008-0538-x. PubMed: 18302013. 18302013 10.1007/s10803-008-0538-x PMC2516538

[27] Hurst RM, Mitchell JT, Kimbrel NA, Kwapil TK, Nelson-Gray RO (2007) Examination of the reliability and factor structure of the autism spectrum quotient (AQ) in a nonclinical sample. Pers Individ Dif 43: 1938–1949. doi:10.1016/j.paid.2007.06.012.

[28] Kloosterman PH, Keefer KV, Kelley EA, Summerfeldt LJ, Parker JDA (2011) Evaluation of the factor structure of the Autism-Spectrum Quotient. Pers Individ Dif 50: 310–314. doi:10.1016/j.paid.2010.10.015.

[29] Lepage JF, Lortie M, Taschereau-Dumouchel V, Théoret H (2009) Validation of French-Canadian versions of the Empathy Quotient and autism spectrum quotient. Can J Behav Sci Rev Can Sci Comportement 41: 272–276.

[30] Lau YP, Gau SSF, Chiu YN, Wu YY, Chou WJ et al. (2013) Psychometric properties of the Chinese version of the Autism Spectrum Quotient (AQ). Res Dev Disabilities 34: 294–305. doi:10.1016/j.ridd.2012.08.005. PubMed: 22985783. 10.1016/j.ridd.2012.08.005 22985783

[31] Wakabayashi A, Baron-Cohen S, Wheelwright S (2006) Are autistic traits an independent personality dimension? A study of the Autism-Spectrum Quotient (AQ) and the NEO-PI-R. Pers Individ Dif 41: 873–883. doi:10.1016/j.paid.2006.04.003.

[32] Baron-Cohen S, Bolton P, Wheelwright S, Short L, Mead G, Smith A, Scahill V (1998) Autism occurs more often in families of physicists, engineers, and mathematicians. Autism 2: 296–301. doi:10.1177/1362361398023008.

[33] Wheelwright S, Baron-Cohen S, Goldenfeld N, Delaney J, Fine D et al. (2006) Predicting Autism Spectrum Quotient (AQ) from the Systemizing Quotient-Revised (SQ-R) and Empathy Quotient (EQ). Brain Res 1079: 47-56. doi:10.1016/j.brainres.2006.01.012. PubMed: 16473340. 16473340 10.1016/j.brainres.2006.01.012

[34] Hurst RM, Nelson-Gray RO, Mitchell JT, Kwapil TR (2006) The relationship of Asperger’s characteristics and schizotypal personality traits in a non-clinical adult sample. J Autism Dev Disord 37: 1711-1720. PubMed: 17149668. 17149668 10.1007/s10803-006-0302-z

[35] Baron-Cohen S (2003) The essential difference. The truth about the male and female brain. New York, NY, USA: Basic Books. 271pp.

[36] Constantino JN, Charman T (2012) Gender bias, female resilience, and the sex ratio in autism. J Am Acad Child Adolesc Psychiatry 51: 756-758. doi:10.1016/j.jaac.2012.05.017. PubMed: 22840545. 22840545 10.1016/j.jaac.2012.05.017

[37] Dworzynski K, Ronald A, Bolton P (2012) How different are girls and boys above and below the diagnostic threshold for autism spectrum disorders? J Am Acad Child Adolesc Psychiatry 51: 788-797. doi:10.1016/j.jaac.2012.05.018. PubMed: 22840550. 22840550 10.1016/j.jaac.2012.05.018

[38] Krahn TM, Fenton A (2010) The Extreme Male Brain Theory of autism and the potential adverse effects for boys and girls with autism. J Bioethical Inq 9: 93-103. PubMed: 23180205. 10.1007/s11673-011-9350-y 23180205

[39] Hofstede GH (2001) Culture’s consequences: Comparing values, behaviors, institutions, and organizations across nations. Thousand Oaks, CA: Sage Publications. 596pp.

[40] House RJ, Hanges PJ, Javidan M, Dorfman PW, Gupta V, editors (2004) Culture, leadership, and organizations. The GLOBE Study of 62 societies. Thousand Oaks: Sage Publishing House. 818pp.

[41] Kossakowska Z, Pęczkowska E (2008) W poszukiwaniu wsparcia. Analiza dostępności usług diagnostycznych i terapeutycznych dla dzieci ze spektrum autyzmu In search of support. The analysis of availability of diagnostic and therapeutic services for children from autism spectrum. [In Polish]. Warsaw: Fundacja SYNAPSIS. p. 32.

[42] Lord C, Rutter M, DiLavore PC, Risi S, Gotham K et al. (2012) Autism Diagnostic Observation Schedule, second Edition. (p. ADOS-2), Manual (Part I): Modules 1-4; Torrance CA Western Psychological Services. 446 p.

[43] Rutter M, LeCouteur A, Lord C (2003) Autism Diagnostic Interview-Revised (ADI-R-WPS). Los Angeles, CA: Western. Psychol Serv: 60.

[44] Rutter M, Bailey A, Lord C (2003) Social Communication Questionnaire-WPS (SCQ-WPS). Los Angeles, CA: Western. Psychol Serv: 26.

[45] Wechsler D (1981) Manual for the Wechsler Adult Intelligence Scale- Revised. New York: Psychological Corporation. 156pp.

[46] Brzeziński J, Gaul M, Hornowska E, Jaworowska A, Machowski A, Zakrzewska M (2004) WAIS-R (PL) - Skala Inteligencji Wechslera dla Dorosłych - Wersja Zrewidowana. Renormalizacja 2004. Wars Pracownia Testów Psychologicznych PTP: 220.

[47] Rakotomalala R (2005) TANAGRA: un logiciel gratuit pour l’enseignement et la recherche. Actes de EGC’2005, RNTI-E-32: 697-702.

[48] Guilford JP (1954) Psychometrics methods. New York: McGraw-Hill 597pp. p

[49] Ingersoll B, Hopwood CJ, Wainer A, Donnellan MB (2011) A comparison of three self-report measures of the broader autism phenotype in a non-clinical sample. Journal of Autism and Developmental Disorders 41: 1646-1657. 21331821 10.1007/s10803-011-1192-2

[50] Meng-Chuan L, Lombardo MV, Pasco G, Ruigrok ANV, Wheelwright SJ et al. (2011) *A* *behavioral* comparison of male and female adults with high functioning autism spectrum conditions. PLOS ONE 6: 1-10. 10.1371/journal.pone.0020835 PMC3113855 21695147

